# Prediction models of COVID-19 fatality in nine Peruvian provinces: A secondary analysis of the national epidemiological surveillance system

**DOI:** 10.1371/journal.pgph.0002854

**Published:** 2024-01-29

**Authors:** Wendy Nieto-Gutierrez, Jaid Campos-Chambergo, Enrique Gonzalez-Ayala, Oswaldo Oyola-Garcia, Alberti Alejandro-Mora, Eliana Luis-Aguirre, Roly Pasquel-Santillan, Juan Leiva-Aguirre, Cesar Ugarte-Gil, Steev Loyola

**Affiliations:** 1 Facultad de Salud Pública, Universidad Peruana Cayetano Heredia, Lima, Perú; 2 Universidad Científica del Sur, Lima, Perú; 3 Dirección de Epidemiología e Investigación, Dirección Regional de Salud Lima Provincias, Lima, Perú; 4 Facultad de Medicina, Universidad Peruana Cayetano Heredia, Lima, Perú; 5 Instituto de Medicina Tropical Alexander von Humboldt, Universidad Peruana Cayetano Heredia, Lima, Perú; 6 Department of Epidemiology, School of Public and Population Health, University of Texas Medical Branch, Galveston, Texas, United States of America; Human Sciences Research Council, SOUTH AFRICA

## Abstract

There are initiatives to promote the creation of predictive COVID-19 fatality models to assist decision-makers. The study aimed to develop prediction models for COVID-19 fatality using population data recorded in the national epidemiological surveillance system of Peru. A retrospective cohort study was conducted (March to September of 2020). The study population consisted of confirmed COVID-19 cases reported in the surveillance system of nine provinces of Lima, Peru. A random sample of 80% of the study population was selected, and four prediction models were constructed using four different strategies to select variables: 1) previously analyzed variables in machine learning models; 2) based on the LASSO method; 3) based on significance; and 4) based on a post-hoc approach with variables consistently included in the three previous strategies. The internal validation was performed with the remaining 20% of the population. Four prediction models were successfully created and validate using data from 22,098 cases. All models performed adequately and similarly; however, we selected models derived from strategy 1 (AUC 0.89, CI95% 0.87–0.91) and strategy 4 (AUC 0.88, CI95% 0.86–0.90). The performance of both models was robust in validation and sensitivity analyses. This study offers insights into estimating COVID-19 fatality within the Peruvian population. Our findings contribute to the advancement of prediction models for COVID-19 fatality and may aid in identifying individuals at increased risk, enabling targeted interventions to mitigate the disease. Future studies should confirm the performance and validate the usefulness of the models described here under real-world conditions and settings.

## Introduction

The COVID-19 pandemic has generated a global health, economic, and humanitarian crisis, causing, until July 2021, more than 100 million cases and more than 3.9 million deaths [1]. Fatality from COVID-19 was estimated to be less than 3% during the first months of the pandemic [2]; however, later estimates reported rates between 10 and 48%, which varied between locations (continents and countries) [3]. The characteristics of each location (environmental, economic, structural, etc.) probably contribute to the changes in fatality. For instance, temperature, concomitant epidemics of other respiratory infections (tuberculosis, etc.) [4], the type of viral variant and changes in its prevalence and dispersion, as well as the availability of medical resources mainly influence fatality rates.

In view of the high fatality rates due to COVID-19, the World Health Organization [5] proposed multiple initiatives to promote the creation of predictive fatality models to assist decision-makers in the development of strategies, planning, and design of public policies for the prioritization of the most vulnerable groups. Different studies have used population databases to assess whether clinical, sociodemographic, and laboratory characteristics influence the occurrence of negative outcomes among COVID-19 cases [6–8]. However, due to the differential distribution and representation of specific races/ethnicities [9], characteristics of healthcare systems [10], social vulnerability (which entail higher tendencies of crowding and viral exposure) [11], economic characteristics (which bring about inequalities in healthcare access) [12], geographic factors (climatic factors and altitude levels) [13, 14], distribution of comorbidities [15], and genetic factors that could modify the disease burden and its trends [16], conduct that the usefulness of these prediction models may be limited in contexts other than those in which they were developed [17].

Peru has had a variable and dynamic COVID-19 incidence [18]. The different presentations of the disease, the interventions (pharmaceutical and non-pharmaceutical), the adopted treatment protocols, and the spread and predominance of local viral variants have most likely influenced the COVID-19 incidence. These factors differed significantly from those observed in the United States and England [19], for example, and even amongst countries in the same region [20, 21]. Globally, Peru had the highest number of deaths per 100,000 inhabitants during the first and second COVID-19 waves [22] and, until July 2021, was the fifth country with the highest case-fatality ratio from COVID-19 [1]. A number of studies have reported various factors associated with COVID-19 fatality in the Peruvian population [23–27]; however, their results could be inaccurate and non-representative given multiple limitations such as; low sample size, lack of temporality, and selection bias. This study aimed to build multiple prediction models of COVID-19 fatality using population data from the Peruvian surveillance system.

## Methodology

### Study design and population

A retrospective cohort was conducted using the database of the national epidemiological surveillance system for COVID-19 (NotiWeb) of the "Centro Nacional de Epidemiología, Prevención y Control de enfermedades" (CDC) of Peru.

The national epidemiological surveillance system for COVID-19 includes information on persons reported as suspected, probable, and confirmed symptomatic cases throughout Peru [28]. However, for this study, only anonymized information from confirmed symptomatic cases reported by the Dirección Regional de Salud de Lima Provincias (DIRESA-LIPRO) was included. The cases included in the study were registered between March 1, 2020, and September 30, 2020 –a time in which vaccination against COVID-19 had not yet begun. The definition of a confirmed case used in this study was based on the guidelines issued by the Peruvian Ministry of Health [28]. Cases with nationalities other than Peruvian and cases under 18 years of age were excluded due to their vulnerability and the lower and different fatality rate compared to the adult population [29], respectively. *(S1 Data)*

### Context

The Peruvian epidemiological surveillance system passively identifies COVID-19 cases and monitors them until the disease is resolved or death. Case notification is mandatory for all health institutions. Therefore, according to the policy of the Peruvian Ministry of Health, all suspected or probable cases are monitored by medical personnel and then reported to the surveillance system using the NotiWeb platform [28].

The DIRESA-LIPRO administers the information on COVID-19 cases reported by health institutions located in 128 districts across the nine provinces of the Peruvian department of Lima: Barranca, Cajatambo, Canta, Cañete, Huaral, Huarochirí, Huaura, Oyón and Yauyos *(S1 Fig)*. According to the most recent national data published before 2020 [30], the nine provinces of Lima account for 3.1% of the Peruvian population, which corresponds to approximately 9% of the entire population of the Lima department. Eighty-three percent of the province’ population reside in urban areas.

### Sampling

The cases registered in the surveillance system were enrolled through a non-probabilistic method, including all those reported as suspected or probable cases of COVID-19. Biological specimens were collected from the cases for SARS-CoV-2 testing. Suspected or probable cases that tested positive were reclassified and reported as confirmed cases.

The power of the sample size was estimated using an interactive calculator (https://riskcalc.org/pmsamplesize/) considering that this study performed a secondary analysis of an epidemiological surveillance database and taking into account the Smeden and Riley’s four sample estimation criteria [31]. It was assumed the inclusion of 35 potential parameters in the prediction model, a fatality rate of 11.0% for Latin American countries [3], an expected value of R2 of 0.1, a shrinkage level (a measure of overfitting) of 0.9, and a C-statistic of 0.94 reported in a previous prediction modeling study [7]. The largest sample size obtained was 2,972 cases to reach a power of 80.0%. Considering that the sample size analyzed here exceeded the largest estimated sample size, it is reasonable to consider that the statistical power is adequate to build prediction models.

### Procedure

Data collection was carried out by personnel from healthcare institutions of the nine provinces using a clinical-epidemiological survey. Suspected/probable cases of COVID-19 were identified, mainly, by their care (emergency visits, etc.) in any of the healthcare institutions and, in less frequency, by their communication through a call center (113 Infosalud, 107 EsSalud, etc.), web page, or mobile app of a health institution. Suspected/probable cases that met the definitions proposed by the Peruvian Ministry of Health were registered in the surveillance system [28]. The DIRESA-LIPRO supervised the correct and complete filling out of the clinical-epidemiological surveys and performed daily quality control of the records in the surveillance system.

### Outcome: COVID-19 fatality

The outcome was defined as the death of a confirmed COVID-19 case with the cause of death registered as COVID-19 disease or a related complication (such as respiratory failure due to COVID, organic failure due to COVID, etc.). The information was obtained from the database of the surveillance system until January 2022 and was corroborated with the database of the National Death System of Peru (SINADEF) [32]. Finally, a dichotomous variable ("no" and "yes") was generated.

### Predictor variables

Age (years), sex (female or male), clinical characteristics at enrollment and registration in the surveillance system (fever, cough, etc.), the severity of the disease based on symptoms (with or without symptoms of severity; considering the classification of COVID-19 Treatment Guidelines Panel of the National Health Institute-NIH), comorbidities (hypertension, diabetes, etc.) and the number of comorbidities (none, 1–2, and ≥3) were analyzed as predictors.

### Statistical analysis

The data analysis was performed using Stata v.16 (StataCorp. 2019. Stata Statistical Software: Release 16. College Station, TX: StataCorp LLC.) and Python v.3.4.3. The database was randomly split into two separate datasets. The first dataset, corresponding to 80% of the total population, was used to build the prediction models, while the other dataset, corresponding to the remaining 20%, were used to validate the models.

A descriptive analysis of participants characteristics was performed in the three databases (global, dataset for model building, and dataset for model validation) using absolute and relative frequencies for the categorical variables, and central tendency and dispersion measures for the numerical variables. Bivariate analyses were performed between the outcome and predictors using logistic regressions since this approach is one of the most widespread for building prediction models and one of the easiest for estimating predictive performance [33].

Considering the sample imbalance in the outcome, we considered adjusting the datasets to construct and evaluate the performance of the prediction models [34]. This involved assigning a higher weight to the minority category of death for COVID-19 to increase its value during training [35]. The weighting was incorporated into the regression model using the “*svy”* command.

Four prediction models were built considering different variable selection strategies: strategy 1) using variables reported as predictors in previously published machine learning models [7, 8, 36]; strategy 2) based on the least absolute shrinkage and selection operator (LASSO) method *(S2 Fig)*; strategy 3) based on the significance (p<0.05) observed in the bivariate logistic regression analyses; and a strategy 4) based on a post-hoc approach which comprised variables consistently included in the models created by the first three strategies. For each model, sensitivity (Se.), specificity (Sp.), predictive values (positive and negative), likelihoods (positive and negative), and areas under the curve (AUC) were estimated. Both Se. and Sp. were estimated for a probability cut-off point selected to yield the most balanced performance between the two parameters. The 95% confidence intervals (95%CI) were estimated for all parameters, and the model selection was based on performance (Se., Sp., and AUC) and parsimony (few variables included).

A sensitivity analysis was performed to assess the robustness and consistency of the models under different scenarios, restricting only to cases with a confirmatory diagnosis by RT-PCR and stratifying by pandemic periods (first period: from January to June 2020; second period: from July 2020 to September 2020; and between peaks of incidence of COVID-19: from June 2020 to July 9^th^, 2020 [37]).

### Ethical aspects

This study is a secondary analysis of a previously collected database, so the risks are minimal. The database was obtained from the national epidemiological surveillance system of the Centers for Disease Control and Prevention (CDC) of Peru. The CDC collects information on all patients with suspected and confirmed diagnoses of COVID-19 as part of their mandatory data collection within the national epidemiological surveillance strategy. Permission to access and use the database for scientific purposes was obtained from DIRESA-LIPRO. Although the database contains personal information of the patients, this was only available for the DIRESA-LIPRO. For the author and the secondary analysis, the data was deidentified. Before data analysis, the study protocol was evaluated and approved by the institutional ethics committee of the Universidad Peruana Cayetano Heredia (068-06-22). Finally, this study was registered in the health research projects platform (PRISA: EI00002044).

## Results

The database included a total of 23,742 confirmed cases of COVID-19 with a positive test and symptom onset date up to September 30, 2020, of which 1,404 were excluded for being under 18 years of age, 169 for not having Peruvian nationality, and 71 because they came from a department other than Lima *(S3 Fig)*. After exclusions, the database included a total of 22,098 cases (mean age 45.96 ± 16.82; 53.41% female) eligible for the analysis. The dataset used for the model building and the one used for validation included a sample of 17,678 (mean age 45.99 ± 16.86; 53.25% female) and 4,420 cases (mean age 45.85 ± 16.64; 54.05% female), respectively. *(Table 1)*.

**Table 1:** Characteristics of the COVID-19 confirmed cases in nine provinces of the department of Lima, Peru.

Variables		Full dataset (N = 22 098)	Dataset for the model construction (N = 17 678)	Dataset for the model validation (N = 4 420)
		N (%)	N (%)	N (%)
**Age** *		45.96 ± 16.82	45.99 ± 16.86	45.85 ± 16.64
**Sex**				
	Female	11803 (53.41)	9414 (53.25)	2389 (54.05)
	Male	10295 (46.59)	8264 (46.75)	2031 (45.95)
**Fiscal address**				
	Barranca	3525 (15.95)	2849 (16.12)	676 (15.29)
	Cajatambo	29 (0.13)	23 (0.13)	6 (0.14)
	Canta	259 (1.17)	195 (1.1)	64 (1.45)
	Cañete	7215 (32.65)	5775 (32.67)	1440 (32.58)
	Huaral	4279 (19.36)	3409 (19.28)	870 (19.68)
	Huarochirí	583 (2.64)	464 (2.62)	119 (2.69)
	Huara	5526 (25.01)	4427 (25.04)	1099 (24.86)
	Oyón	143 (0.65)	111 (0.63)	32 (0.72)
	Yauyos	183 (0.83)	142 (0.8)	41 (0.93)
	Outside of the Lima provinces**	356 (1.61)	283 (1.60)	73 (1.65)
**Diagnostic test**				
	RT-PCR	4291 (19.44)	3454 (19.56)	837 (18.95)
	Serological test	17756 (80.45)	14183 (80.33)	3573 (80.91)
	Antigen test	25 (0.11)	19 (0.11)	6 (0.14)
**Notifying institution**				
	Ministry of Health (MINSA)	19754 (89.39)	15795 (89.35)	3959 (89.57)
	Social Health Insurance (EsSalud)	1315 (5.95)	1061 (6)	254 (5.75)
	Other (private clinics, etc.)	1029 (4.66)	822 (4.65)	207 (4.68)
**Symptoms at the enrolment**				
	Fever	9238 (41.8)	7368 (41.68)	1870 (42.31)
	General discomfort	11356 (51.39)	9111 (51.54)	2245 (50.79)
	Cough	14450 (65.39)	11588 (65.55)	2862 (64.75)
	Sore throat	13012 (58.88)	10385 (58.75)	2627 (59.43)
	Nasal congestion	5898 (26.69)	4714 (26.67)	1184 (26.79)
	Dyspnea	5321 (24.08)	4305 (24.35)	1016 (22.99)
	Diarrhea	2690 (12.17)	2146 (12.14)	544 (12.31)
	Nausea and vomiting	1742 (7.88)	1401 (7.93)	341 (7.71)
	Headache	8676 (39.26)	6884 (38.94)	1792 (40.54)
	Confusion	239 (1.08)	182 (1.03)	57 (1.29)
	Muscle pain	4952 (22.41)	3940 (22.29)	1012 (22.9)
	Abdominal pain	591 (2.67)	455 (2.57)	136 (3.08)
	Chest pain	2987 (13.52)	2356 (13.33)	631 (14.28)
	Joint pain	648 (2.93)	522 (2.95)	126 (2.85)
	Dysosmia and dysgeusia	982 (4.44)	786 (4.45)	196 (4.43)
	Earache	12 (0.05)	9 (0.05)	3 (0.07)
**Severity by symptoms**				
	No symptoms of severity	18613 (85.56)	14935 (85.79)	3678 (84.61)
	With severe symptoms	3142 (14.44)	2473 (14.21)	669 (15.39)
**Comorbidities**				
	Cardiovascular disease	1574 (7.12)	1278 (7.23)	296 (6.7)
	Hypertension	396 (1.79)	321 (1.82)	75 (1.7)
	Dyslipidemia	77 (0.35)	68 (0.38)	9 (0.2)
	Diabetes	1133 (5.13)	883 (4.99)	250 (5.66)
	Thyroid disease	163 (0.74)	121 (0.68)	42 (0.95)
	Liver disease	115 (0.52)	91 (0.51)	24 (0.54)
	Neurological disease	124 (0.56)	100 (0.57)	24 (0.54)
	Immunodeficiency	35 (0.16)	29 (0.16)	6 (0.14)
	Kidney disease	104 (0.47)	91 (0.51)	13 (0.29)
	Lung disease	242 (1.1)	189 (1.07)	53 (1.2)
	Asthma	343 (1.55)	275 (1.56)	68 (1.54)
	Cancer	111 (0.5)	89 (0.5)	22 (0.5)
	Obesity	863 (3.91)	691 (3.91)	172 (3.89)
	Tuberculosis	145 (0.66)	120 (0.68)	25 (0.57)
**Number of comorbidities**				
	None	17817 (80.63)	14254 (80.63)	3563 (80.61)
	1–2	4129 (18.68)	3303 (18.68)	826 (18.69)
	> 2	152 (0.69)	121 (0.68)	31 (0.7)
**Death**				
	No	20433 (92.47)	16346 (92.47)	4087 (92.47)
	Yes	1665 (7.53)	1332 (7.53)	333 (7.53)

*Mean ± standard deviation

**The person resides in one of the nine provinces of Lima, but his/her fiscal address was outside of Lima

The sociodemographic characteristics, the type of test used for diagnosis, the notifying healthcare institution, clinical characteristics and fatality were comparable within datasets *(Table 1)*. Also, survival curves within datasets were comparable *(S4 Fig)*.

Initially, three models were built using the following strategies; strategy 1 (includes variables previously described as predictors elsewhere) with 13 variables, strategy 2 (based on the LASSO method) with nine variables, and strategy 3 (based on significance in the bivariate logistic regression) with 22 variables *(Table 2)*. Interestingly, the three models consistently included three variables: age, sex, and dyspnea *(Table 2)*. In view of this, we defined a fourth strategy to build a post-hoc model including only the previous three variables.

**Table 2:** Predictive factors of fatality among COVID-19 confirmed cases using the model-building dataset (N = 17 678).

Variables		Bivariate analysis	Models			
			Strategy 1	Strategy 2	Strategy 3	Strategy 4
		OR (95%CI)	OR (95%CI)	OR (95%CI)	OR (95%CI)	OR (95%CI)
**Age**		**1.09 (1.09–1.10)**	**1.08 (1.08–1.09)**	**1.08 (1.08–1.09)**	**1.08 (1.07–1.09)**	**1.08 (1.08–1.09)**
**Sex**						
	Female	Ref.	Ref.	Ref.	Ref.	Ref.
	Male	**2.23 (1.99–2.51)**	**1.82 (1.55–2.13)**	**1.71 (1.46–2.00)**	**1.80 (1.53–2.11)**	**1.78 (1.52–2.08)**
**Symptoms at the enrolment**						
	Fever	**1.43 (1.29–1.61)**	1.11 (0.95–1.30)	–	1.09 (0.92–1.28)	–
	General discomfort	**1.71 (1.53–1.93)**	–	**1.41 (1.20–1.66)**	**1.43 (1.22–1.68)**	–
	Cough	**2.31 (2.01–2.66)**	–	**1.55 (1.29–1.88)**	**1.62 (1.34–1.96)**	–
	Sore throat	**0.78 (0.70–0.88)**	–	–	0.96 (0.81–1.12)	–
	Nasal congestion	**0.73 (0.63–0.83)**	–	–	0.91 (0.76–1.09)	–
	Dyspnea	**9.87 (8.71–11.18)**	**7.36 (6.32–8.57)**	**7.14 (6.12–8.33)**	**6.96 (5.96–8.14)**	**7.59 (6.53–8.81)**
	Diarrhea	0.93 (0.78–1.11)	–	–	–	–
	Nausea and vomiting	1.20 (0.99–1.46)	–	–	–	–
	Headache	**0.57 (0.51–0.65)**	–	**0.61 (0.51–0.72)**	0.59 (0.50–0.70)	–
	Confusion	**3.53 (2.48–5.04)**	–	–	**2.81 (1.36–5.83)**	–
	Muscle pain	1.01 (0.88–1.15)	–	–	–	–
	Abdominal pain	1.02 (0.72–1.45)	–	–	–	–
	Chest pain	1.13 (0.96–1.32)	–	–	–	–
	Joint pain	**1.40 (1.05–1.87)**	–	–	0.91 (0.63–1.31)	–
	Dysosmia and dysgeusia	**0.09 (0.04–0.20)**	–	**0.19 (0.79–0.45)**	–	–
	Earache	1.53 (0.19–12.28)	–	–	–	–
**Severity by symptoms**						
	No symptoms of severity	Ref.		Ref.	Ref.	
	With severe symptoms	**1.33 (1.15–1.54)**	–	1.14 (0.93–1.39)	1.14 (0.93–1.40)	–
**Comorbidities**						
	Cardiovascular disease	**3.63 (3.13–4.22)**	1.25 (0.97–1.61)	–	0.99 (0.70–1.39)	–
	Hypertension	**2.72 (2.03–3.65)**	0.89 (0.55–1.44)	–	0.72 (0.43–1.20)	–
	Dyslipidemia	0.37 (0.09–1.52)	–	–	–	–
	Diabetes	**2.54 (2.10–3.06)**	**1.54 (1.17–2.02)**	–	1.15 (0.81–1.63)	–
	Thyroid disease	0.87 (0.42–1.78)	–	–	–	–
	Liver disease	**2.64 (1.53–4.54)**	–	–	0.90 (0.37–2.24)	–
	Neurological disease	**2.91 (1.76–4.80)**	–	–	1.21 (0.42–3.51)	–
	Immunodeficiency	0.91 (0.22–3.83)	4.45 (0.74–26.75)	–	–	–
	Kidney disease	**4.99 (3.15–7.89)**	**3.10 (1.48–6.52)**	–	**2.93 (1.42–6.06)**	–
	Lung disease	**2.94 (2.04–4.25)**	1.37 (0.78–2.39)	–	0.97 (0.53–1.78)	–
	Asthma	0.91 (0.57–1.45)	1.14 (0.57–2.28)	–	–	–
	Cancer	**2.30 (1.30–4.09)**	1.16 (0.52–2.57)	–	0.79 (0.35–1.81)	–
	Obesity	1.29 (0.99–1.67)	**1.93 (1.36–2.74)**	**1.88 (1.32–2.68)**	–	–
	Tuberculosis	0.99 (0.50–1.97)	–	–	–	–
**Number of comorbidities**						
	None	Ref.			Ref.	
	1–2	**2.60 (2.31–2.94)**	–	–	**1.51 (1.12–2.03)**	–
	>2	**5.03 (3.30–7.69)**	–	–	2.09 (0.84–5.20)	–

*Strategy 1: variables included since they were previously reported as predictors of death in other studies (References 7, 8, 27); Strategy 2: variables included by the LASSO method; Strategy 3: variables included according to their statistical significance in bivariate analysis; Strategy 4: model created post-hoc with variables that were consistently included in the three previously constructed models.

Upon observing this pattern, a post-hoc strategy was implemented. When evaluating the performance of the constructed models, it was identified that for a death probability cutoff of 52%, similar estimates were obtained, with slightly higher sensitivity and specificity for strategy 3 (Sensitivity (S): 83.08%; Specificity (E): 82.30%), followed by strategy 1 (S: 82.58%; E: 82.07%), strategy 2 (S: 80.00%; E: 82.35%), and strategy 4 (S: 80.78%; E: 81.75%) *(Table 3)*. Similarly, similar areas under the curve were observed for the models obtained through the four variable selection strategies *(S5 Fig)*.

**Table 3:** Performance of the four prediction models for death in COVID-19 confirmed cases (N = 4 420).

Performance	Strategy 1	Strategy 2	Strategy 3	Strategy 4
	Estimate (95%CI)	Estimate (95%CI)	Estimate (95%CI)	Estimate (95%CI)
Sensitivity*	82.58%	80.06%	83.08%	80.78%
Specificity*	82.07%	82.35%	82.30%	81.75%
Area under the curve (AUC)	0.89 (0.87–0.91)	0.88 (0.87–0.90)	0.89 (0.87–0.91)	0.88 (0.86–0.90)
Positive likelihood	4.60	4.54	4.69	4.43
Negative likelihood	0.21	0.24	0.21	0.24

*Cut-off point of the probability of the outcome: 52%

**Fig 1:**
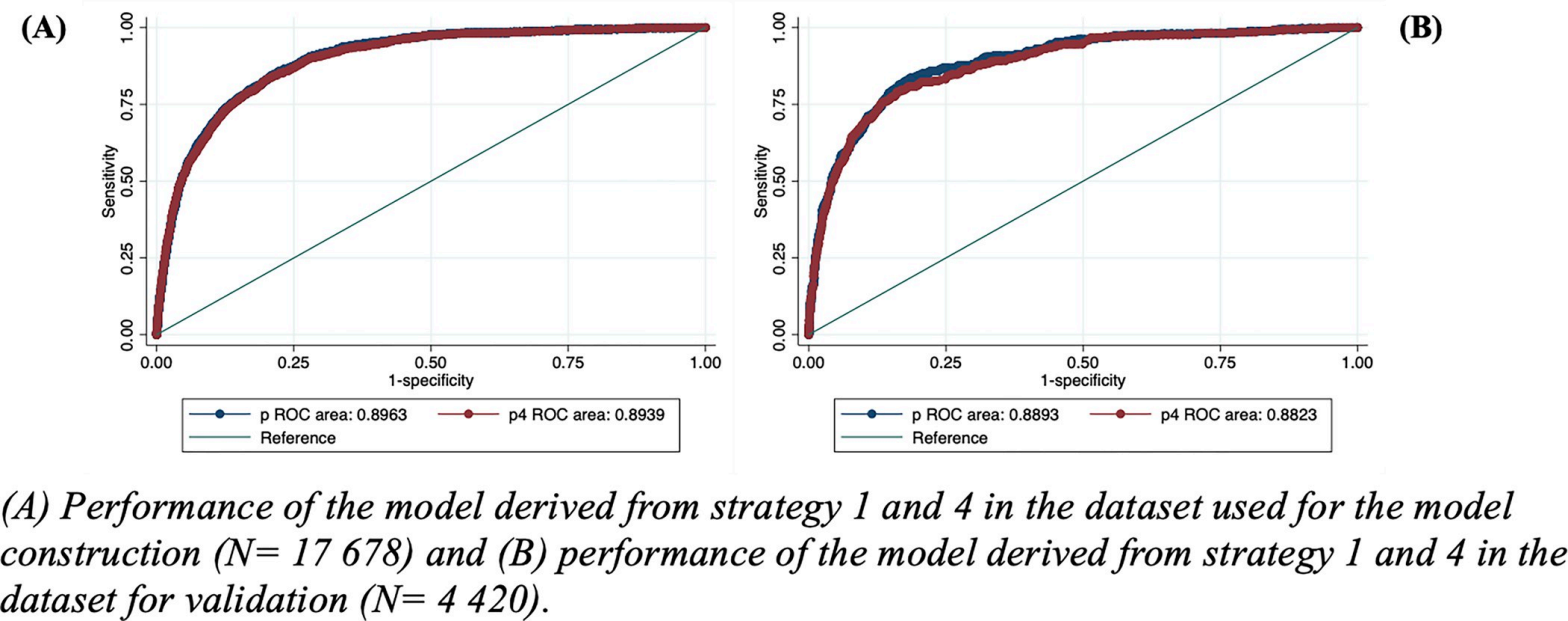
Validation of the selected models for the prediction of death in confirmed cases of COVID-19. (A) Performance of the model derived from strategy 1 and 4 in the dataset used for the model construction (N = 17 678) and (B) performance of the model derived from strategy 1 and 4 in the dataset for validation (N = 4 420).

Although the four variable selection strategies resulted in models with similar performances, models constructed with strategies 1 and 4 were selected for the following reasons. The model from strategy 1 included a smaller number of variables compared to strategy 3 (13 vs. 22), and in comparison, to strategy 2, the performance was slightly better (AUC 0.89 vs. 0.88). On the other hand, the model from strategy 4 is a condensed model with performance comparable to the other strategies. Interestingly, the area under the curve of the selected models in the validation and creation datasets was comparable for the selected model strategies (Strategy 1: 0.89 vs. 0.89 / Strategy 4: 0.89 vs. 0.88; Fig 1). Moreover, using the validation dataset, the performance of the models from strategies 1 and 4 remained stable when conducting a sensitivity analysis including only cases confirmed by RT-PCR (Strategy 1: 0.89 vs. 0.89 / Strategy 4: 0.86 vs. 0.88) and according to pandemic periods (Strategy 1: first period = 0.89 vs. 0.89; second period = 0.89 vs. 0.89; peaks between incidence = 0.88 vs. 0.89 / Strategy 4: first period = 0.88 vs. 0.88; second period = 0.90 vs. 0.88; peaks between incidence = 0.87 vs. 0.88) *(S6 Fig)*.

## Discussion

Throughout the pandemic, Peru was one of the countries with the highest number of deaths from COVID-19 per 100,000 inhabitants and with a case fatality rate of approximately 5%, which was higher than countries such as Mexico and the United States [38]. Fatality has been concentrated mainly in the city of Lima, the capital of Peru, because most the population were concentrated there [1]. Lima is surrounded by nine provinces; unfortunately, to the best of our knowledge, despite the great connectivity between the capital and the provinces, fatality and its trends in these provinces have not been explored. The disparities between the city center of Lima and its provinces can be substantial, primarily driven by cultural, ethnic, social, racial, economic, and resource inequalities factors [39], that may contribute to the variations in COVID-19 trends between these locations. In this regard, the results of this study provide an initial characterization of this population. Specifically, our study explored various predictors of death from COVID-19 in the nine provinces of the department of Lima and identified a fatality of 7.5%, corresponding to the first and second pandemic waves. Overall, the high fatality can be attributed to the low preparedness of the Peruvian health system to prevent fatalities. Notably, Peruvian provinces faced much greater challenges due to their limited number of high-capacity hospitals and thus faced a pandemic not only with an unprepared system but also with shortages of intensive care units, medical devices, and specialized personnel [22]. On the other hand, it is necessary to consider that Peru also prioritized the use of serological or rapid tests for the diagnosis of COVID-19, as reflected in our study. This approach resulted in a missed opportunity to diagnosed early and, consequently, in a hindered containment of future infections and prevention of fatalities, especially in provinces–such as those described here–with no available laboratories to perform molecular testing.

Considering that the COVID-19 pandemic was characterized by the disproportionate use of health resources, it is still necessary to structure and articulate prioritization systems to prevent the occurrence of negative outcomes in vulnerable populations. Here, we describe the development of four models to predict death by COVID-19 considering different variable selection strategies. Globally, all models performed adequately; however, based on the criteria described above, we choose the models derived from strategies 1 and 4. Nevertheless, the use of the other two models remains possible and therefore, we recommend further evaluation.

The model constructed with strategy 1 included clinical predictors reported in previous studies [7, 8, 36]. These studies used population-based data from the United States and described models that performed well (AUC = 0.8) [7, 8, 36]. Although we hypothesized that using previously described models in populations other than those for which they were constructed might not be appropriate because of potential different characteristics between populations, health systems, or the epidemiology of COVID-19; we observed that the use of the variables previously described generates a model that performs well for the prediction of COVID-19 fatality in the Peruvian population studied. We even reported an AUC greater than 0.8, which was robust in the validation and sensitivity analysis. Also, the performance described here is comparable to that reported in other studies that use national databases [7, 8, 36] and clinical [7, 36] and laboratory [8] information. However, although the clinical variables of our model were obtained at patient enrollment, is likely that other symptoms prior to care or symptoms that appeared during the clinical course play an important role in the prediction. On the other hand, we considered some variables as a proxy of unmeasured variables. For instance, fever and hypertension were considered as proxies for temperature and arterial pressure at enrollment [7, 8, 36]. Despite this, we consider that this constraint does not significantly affect the performance of the model, due to the close relationship of the proxies used with the unmeasured variables. On the other hand, since the comorbidities were obtained through self-reports, and given that it is conceivable that there may be an underlying under-diagnosis of comorbidities, it is therefore likely that their effect and contribution to the model are underestimated, and consequently, the model performance is underestimated.

The model built based on strategy 4 only comprised three variables that were consistently included in the other three models. Although this model was the most parsimonious and performed similarly to the model constructed with strategy 1, it is important to note that it was built post-hoc and was based on findings from the other models. Therefore, the model 4 could be affected by multiple limitations described elsewhere [40]. It is important to note that the model was robust in sensitivity analyses, and its performance was comparable with other non-complex [10, 41, 42] and complex models described elsewhere [7, 8, 36]. Therefore, based on these observations, we consider that the model derived from strategy 4 could be used with caution.

In order to opt for one of the selected models, we propose a prior assessment of the quality of the data. As an example, if there is no confidence in how the information on comorbidities was measured or collected, the model 4 could be used to predict COVID-19 death. On the other hand, if the quality of the data related to comorbidities is reliable, model 1 could be the most suitable for predicting death, considering that the better marginal performance over model 4 would translate into better predictive power.

The selected models included some variables that were significantly associated with the outcome, such as dyspnea, age, male gender, diabetes, kidney disease, and obesity. These observations are discussed below.

Dyspnea was one of the characteristics that most increased the possibility of death, approximately 7-fold. Artificial intelligence modeling identified dyspnea as one of the most predictive factors of COVID-19 fatality [7]. This is because severe stages of COVID-19 are characterized by hypoxia, which often leads to respiratory failure and causes dyspnea. Previous studies have reported a higher incidence of death as oxygen saturation decreases [43, 44], which has also been corroborated in the Peruvian population [23, 24, 26].

In this study we identified that the higher the age, the greater the possibility of death. This relationship has been previously described [45–47], specifically indicating that fatality of COVID-19 increases by almost 50% in older age groups [47]. This is likely due to the fact that the burden of disease increases in older population, given that increased probability of comorbidities, polypharmacy, and frailty, and consequently a greater risk of negative health outcomes develop. It is important to consider that the relationship between age and death may be different in settings where vaccination coverage is high in the elderly and infection rates are higher in the young adult population [47]. Therefore, the extrapolation of this finding must be carried out with caution.

The chance of death from COVID-19 was approximately doubled in males. Previous studies have suggested that females have a better understanding of their health and potentially quicker access to healthcare compared to males, reducing the probability of death [48]. However, recent studies conducted in the context of COVID-19 have identified that females experience a higher delay in accessing healthcare [49, 50]. Thus, the association may not be related to delayed access, which is understandable given the fear surrounding the disease at the beginning of the pandemic. On the other hand, some studies hypothesized that sex hormones influence the response against COVID-19, suggesting that there is a higher expression of immune-related genes in women, which leads to a higher antibody response and protection against infections [51]. Recently, it has been reported that women maintain a high immune reactivity after viral infections and generate higher humoral responses to vaccines compared with men [52]. For this reason, if we consider the extrapolation of this observation in scenarios with variable COVID-19 vaccine coverage or infection, it would be expected that this association would remain.

Comorbidities such as diabetes, kidney disease, and obesity increased the chance of death from COVID-19. Although, it is known that COVID-19 causes myelopoiesis, T cells and natural killers dysregulation, and uncontrolled production of cytokines; the extent to which underlying comorbidity influences the immune response in the SARS-CoV-2 infection remains unknown [53]. However, previous studies have reported that obesity and diabetes lead to dysregulation of immune cells and hormones, generating decreased immunity and a higher probability of hyperinflammation [53–55], which consequently translates into a boosting of infection and the development of multiple adverse health events. On the other hand, regarding chronic kidney disease, it is known that normal renal function contributes to immune homeostasis given the filtration of circulating cytokines and immunogenic pathogenic components and, thus, inflammation is limited [56]. In this instance, decreased renal function leads to increased activation of innate immune cells and increased cytokine production [57], and the subsequent cytokine storm, thereby generating an exponentiation of the disease severity [53]. Although previous studies have described the relationship between other comorbidities and death [53, 58–60], the uncertainty in the measurement of comorbidities may play an important role in the non-association found.

### Implications for clinical practice

Currently, various clinical practice guidelines for COVID-19, especially those in low and middle-income countries, incorporate prediction models for assessing fatality risk and disease prognosis. These models help prioritize vulnerable groups for treatments, hospitalization, etc., considering their limited resources [61]. However, these guidelines often include laboratory variables and are primarily based on data from high-income countries. In contrast, our model relies solely on clinical and sociodemographic variables. This makes it suitable for use in clinical practices where laboratory tests are limited or unavailable, presenting a feasible tool, for example, in primary care services. By doing so, we can aid in the identification of prioritized groups for transfer to more complex centers or for continuous disease monitoring. Furthermore, our model’s validation in a Peruvian population enhances the certainty of evidence, facilitating its extrapolation for decision-making in other countries with similar healthcare characteristics.

### Limitation and strengths

This study is subject to various limitations. First, due to the fact that only the information recorded in the surveillance system was analyzed, the fatality of COVID-19 may be underestimated since it did not include information on: unreported cases, cases that did not seek healthcare, cases identified but not registered in the system by notifiers, or cases that were not confirmed due to a lack of diagnostic tests. Therefore, the extrapolation of the models only applies to symptomatic individuals with confirmatory tests for COVID-19. Secondly, given that in Peru serological tests detecting antibodies were implemented as diagnostic for COVID-19, our results could be affected by misclassification associated with the use of a diagnostically limited test. However, to address this limitation, a sensitivity analysis was conducted that included only cases diagnosed by RT-PCR. This evaluation suggested that, despite the existence of misclassification, the models’ performance was robust in the analyzed population subgroup. Thirdly, the models constructed in this study only take into account individuals’ first infection to avoid multiple measurements of the infection, excluding the possibility of analyzing the number of reinfections as a predictive variable. Future studies should verify whether this variable would substantially influence in the prediction models performance. Four, because the surveillance system lacked other sociodemographic (i.e socioeconomic status) and laboratory information such as leukocytes, blood group, platelets, D-dimer, among others, their evaluation within the prediction models was not possible. However, even if previous prediction models incorporated these variables, they have not significantly contributed to improving performance, yielding estimates comparable to models that include only clinical variables [62]. Therefore, their contribution in a context of limited healthcare resources may not be beneficial due to feasibility issues. Similarly, variables related to healthcare resources (e.g., the number of available hospital beds, etc.) were not considered within the models, even though they could be related to the outcome. While this data might have been accessible during the period when this population was enrolled, considering that data on available healthcare resources during the pandemic were freely accessible, currently, this report is not updated, and obtaining it might not be feasible. Therefore, its inclusion would complicate a model intended to be applied in different clinical practice contexts. Also, considering that the performance of the models built in this study reports areas under the curve ranging between 80%, we believe that the contribution of this variable would not be substantially important. Fifth, the search for a better healthcare system, such as in the capital of the Lima department, could have led to the displacement of cases to other locations not analyzed. Therefore, these cases may not have been reported to the DIRESA-LIPRO, nor included in the analyses. Nevertheless, given the mobility restrictions implemented, particularly during the first pandemic wave, it is possible that if there were any movements, they were minimal and did not significantly affect our results.

Finally, considering that the data were collected during the early periods of the COVID-19 pandemic, the extrapolation of results to the current context (different viral variants and vaccination) should be cautious. However, while we would expect the model’s performance to be affected in these contexts, we would not expect a difference in the variables included in this study. Moreover, even in previous studies in contexts with a predominance of other viral variants, current evidence indicates a change in fatality trends depending on the type of variant [63], but not a change in the known predictive factors since the beginning of the pandemic [64, 65].

Despite the described limitations, to our knowledge, this study is one of the few that has explored multiple variable selection strategies for creating predictive models for a Latin American population. It is also one of the few that has validated models previously created in other contexts at a population level. Additionally, this study analyzes data derived from a specific system that includes a population from provinces surrounding the capital of the country, possessing characteristics different from the capital but similar to rural areas. Overall, the study contributes to current knowledge, increasing the certainty of evidence, primarily in terms of precision and direct evidence, to be applied in decision-making in similar contexts. Finally, we present an exercise that can be used for other scenarios similar to the COVID-19 pandemic, allowing for the creation of locally validated models.

## Conclusion

Herein, we describe four models with different variable selection strategies for the prediction of COVID-19 fatality. Our findings suggest that, regardless of the strategy, the models displayed comparable performances. However, two prediction models, whose performances were optimal in the validation and sensitivity analyses, exhibited slight outperformance due to their plausibility with the outcome and/or their parsimony. Future studies should corroborate the performance and validate the usefulness of the models described under both actual and real-world conditions.

## Supporting information

S1 Data Database of the study. (DTA)

S1 Fig Distribution of the total population in the nine provinces of Lima-Peru. Map source: Instituto Geográfico Nacional of Peru–Year 2018. *The map corresponds to the department of Lima-Peru. **The data regarding the distribution of the population in the provinces of Lima were obtained from the latest information reported by the National Center for Epidemiology, Prevention, and Control of Diseases of Peru in 2016. ***The percentage corresponds to the distribution with respect to the total population of Peru.
(TIF)

S2 Fig Results of the selection of variables using the LASSO method. *Glossary: Age (x0); Fever (x1); general discomfort (x2); cough (x3); sore throat (x4); nasal congestion (x5); respiratory distress (x6); diarrhea (x7); vomiting (x8); headache (x9); confusion (x10); muscle pain (x11); abdominal pain (x12); chest pain (x13); joint pain (x14); dyssomnia or dysgeusia (x15); ear pain (x16); pregnancy (x17); abortion (x18); cardiovascular disease (x19); hypertension (x20); dyslipidemia (x21); diabetes (x22); thyroid disease (x23); liver disease (x24); neurological disease (x25); immunodeficiency (x26); kidney disease (x27); lung disease (x28); asthma (x29); cancer (x30); obesity (x31); tuberculosis (x32); sex (x33); severity according to symptoms (x34); number of comorbidities (x35).
(TIF)

S3 Fig COVID-19 case selection flowchart. (TIF)

S4 Fig Survival curves of confirmed cases of COVID-19 in nine provinces of Lima, Peru. Overall survival of cases in the full dataset (A), in the dataset used for model building (B), and in the dataset used for the validation (C).
(TIF)

S5 Fig ROC curves of the prediction models. *The specified values of areas under the curve correspond to the following models: 1) "p ROC area": Strategy 1; 2) "p2 ROC area": Strategy 2; 3) "p3 ROC area": Strategy 3; 4) "p4 ROC area": Strategy 4.
(TIF)

S6 Fig Sensitivity analysis of the performance of the selected models in the dataset for validation. 1. Validation of the model for Strategy 1 (A) in cases confirmed by RT-PCR and in confirmed cases reported during (B) the first period and (C) the second period of the pandemic; 2. Validation of the model for Strategy 4 (D) in cases confirmed by RT-PCR and in confirmed cases reported during (E) the first period and (F) the second period of the pandemic; 3. Validation of the model for Strategy 1 in the period between peaks of COVID-19 incidence (G) and (H) validation of the model for Strategy 4 in the period between peaks of COVID-19 incidence.
(TIF)
