# Supplementary figures and images for: Prediction models of COVID-19 fatality in nine Peruvian provinces: A secondary analysis of the national epidemiological surveillance system
Source: PLOS Glob Public Health. 2024 Jan 29;4(1):e0002854. doi: 10.1371/journal.pgph.0002854 (PMC10824411; doi:10.1371/journal.pgph.0002854)

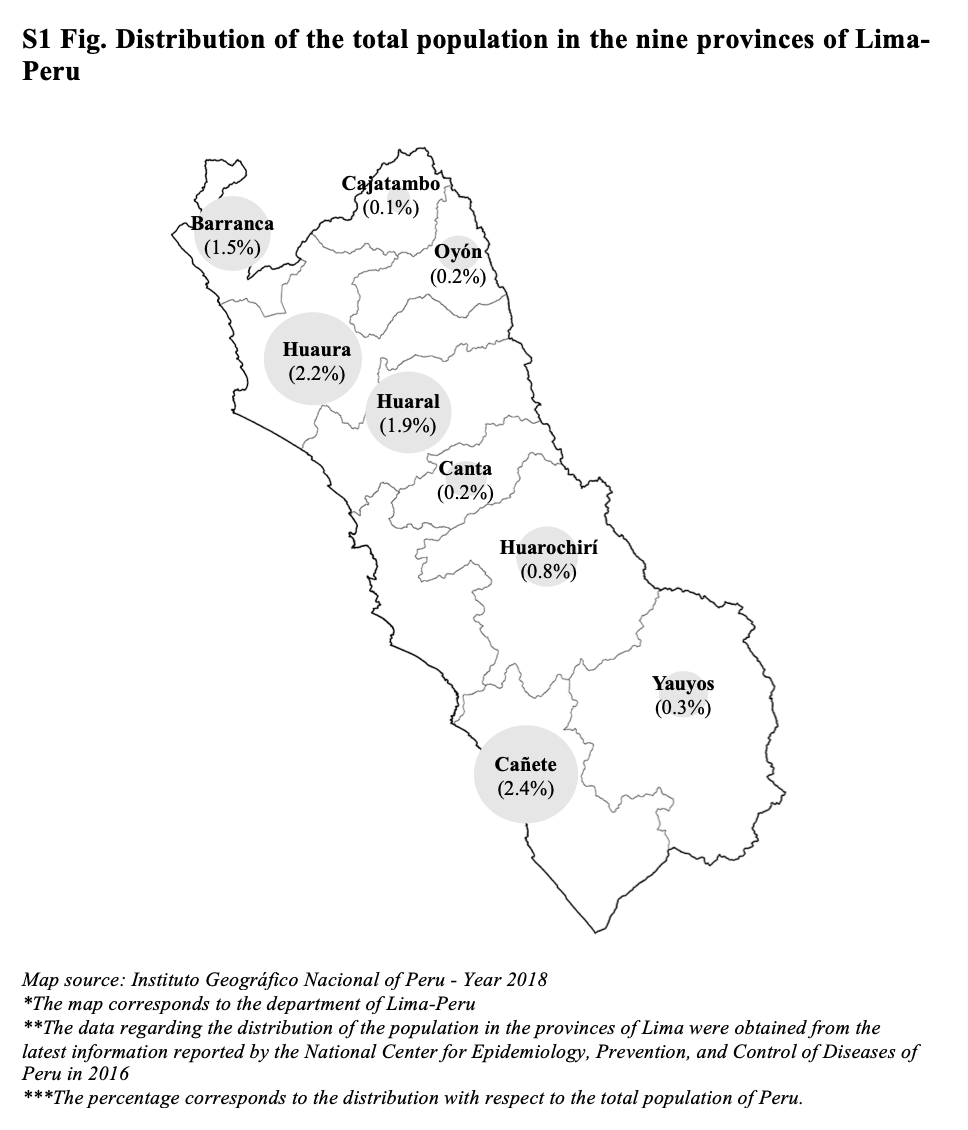

Supplement: S1 Fig — Map source: Instituto Geográfico Nacional of Peru–Year 2018. *The map corresponds to the department of Lima-Peru. **The data regarding the distribution of the population in the provinces of Lima were obtained from the latest information reported by the National Center for Epidemiology, Prevention, and Control of Diseases of Peru in 2016. ***The percentage corresponds to the distribution with respect to the total population of Peru. (TIF) [file pgph.0002854.s002.tif]

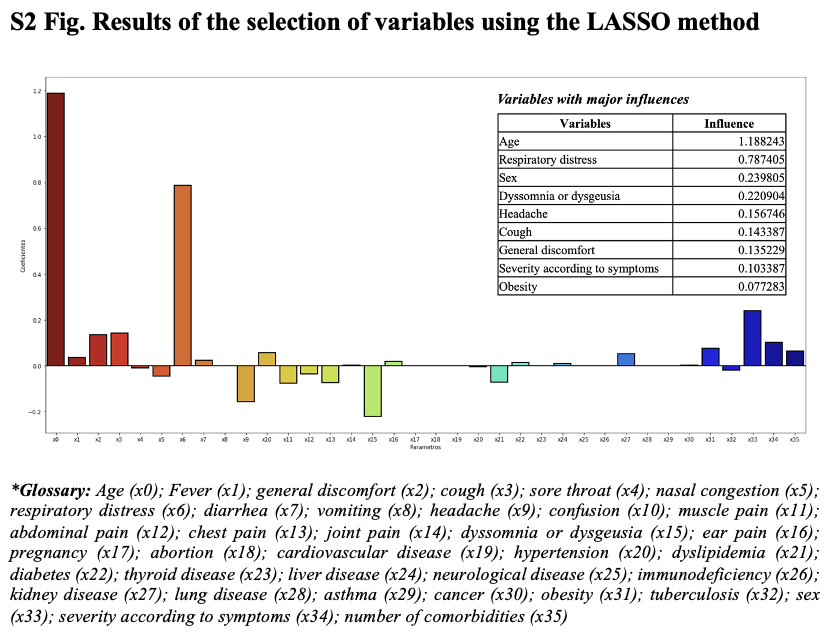

Supplement: S2 Fig — *Glossary: Age (x0); Fever (x1); general discomfort (x2); cough (x3); sore throat (x4); nasal congestion (x5); respiratory distress (x6); diarrhea (x7); vomiting (x8); headache (x9); confusion (x10); muscle pain (x11); abdominal pain (x12); chest pain (x13); joint pain (x14); dyssomnia or dysgeusia (x15); ear pain (x16); pregnancy (x17); abortion (x18); cardiovascular disease (x19); hypertension (x20); dyslipidemia (x21); diabetes (x22); thyroid disease (x23); liver disease (x24); neurological disease (x25); immunodeficiency (x26); kidney disease (x27); lung disease (x28); asthma (x29); cancer (x30); obesity (x31); tuberculosis (x32); sex (x33); severity according to symptoms (x34); number of comorbidities (x35). (TIF) [file pgph.0002854.s003.tif]

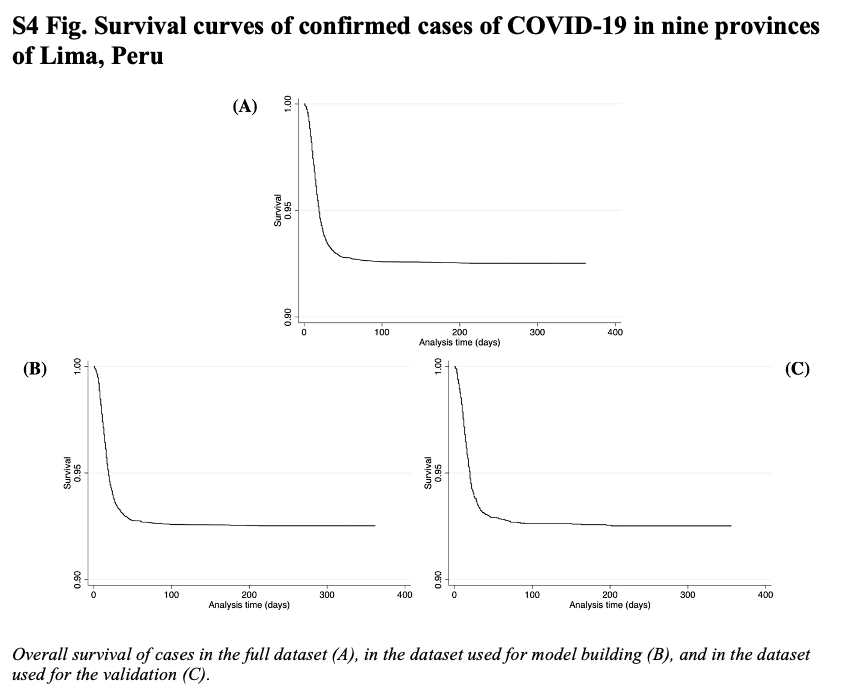

Supplement: S4 Fig — Overall survival of cases in the full dataset (A), in the dataset used for model building (B), and in the dataset used for the validation (C). (TIF) [file pgph.0002854.s005.tif]

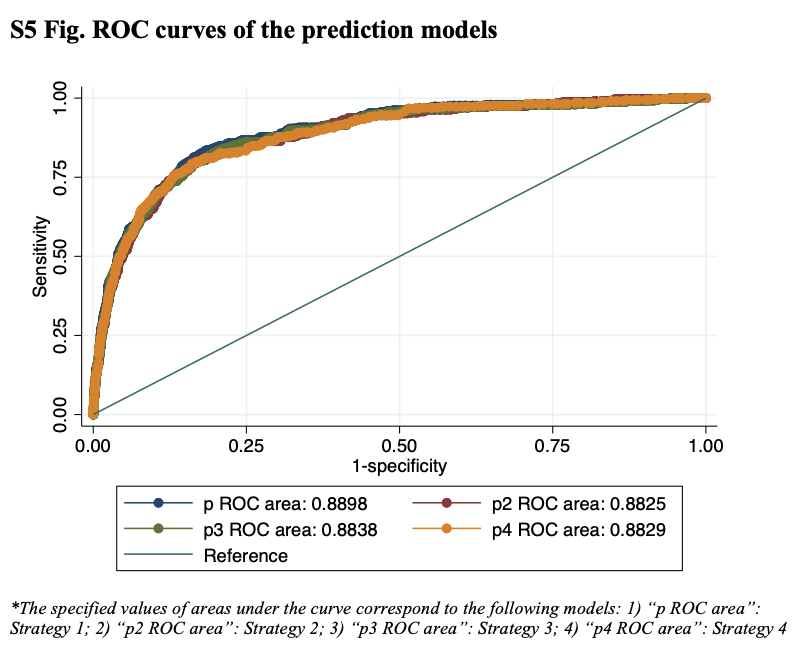

Supplement: S5 Fig — *The specified values of areas under the curve correspond to the following models: 1) "p ROC area": Strategy 1; 2) "p2 ROC area": Strategy 2; 3) "p3 ROC area": Strategy 3; 4) "p4 ROC area": Strategy 4. (TIF) [file pgph.0002854.s006.tif]

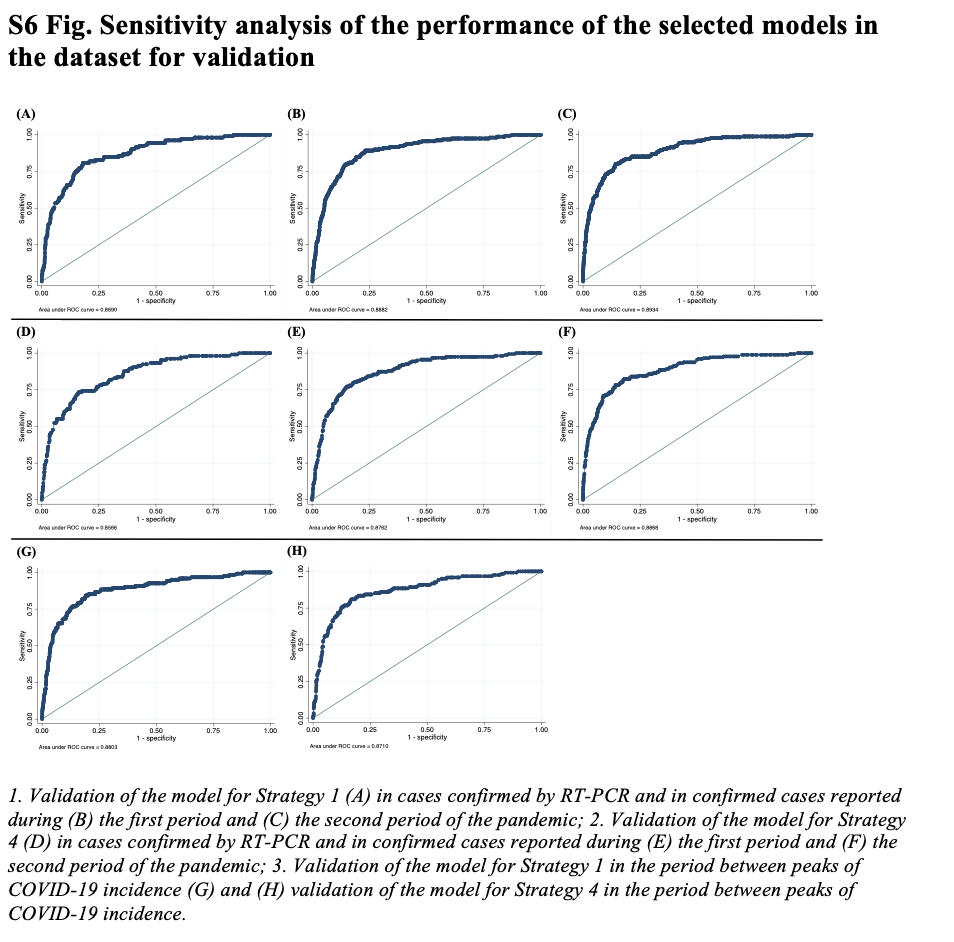

Supplement: S6 Fig — 1. Validation of the model for Strategy 1 (A) in cases confirmed by RT-PCR and in confirmed cases reported during (B) the first period and (C) the second period of the pandemic; 2. Validation of the model for Strategy 4 (D) in cases confirmed by RT-PCR and in confirmed cases reported during (E) the first period and (F) the second period of the pandemic; 3. Validation of the model for Strategy 1 in the period between peaks of COVID-19 incidence (G) and (H) validation of the model for Strategy 4 in the period between peaks of COVID-19 incidence. (TIF) [file pgph.0002854.s007.tif]
